# CAPI-Detect: machine learning in capillaroscopy reveals new variables influencing diagnosis

**DOI:** 10.1093/rheumatology/keaf073

**Published:** 2025-02-07

**Authors:** Gema M Lledó-Ibáñez, Luis Sáez Comet, Mayka Freire Dapena, Miguel Mesa Navas, Miguel Martín Cascón, Alfredo Guillén del Castillo, Carmen Pilar Simeon, Elena Martinez Robles, José Todolí Parra, Diana Cristina Varela, Génesis Maldonado, Adela Marín, Laura Pérez Abad, Jimena Aramburu, Laura Vela, Eduardo Ramos Ibáñez, Borja del Carmelo Gracia Tello

**Affiliations:** Department of Autoimmune Diseases, Institut Clinic de Medicina i Dermatologia, Hospital Clínic de Barcelona, Barcelona, Spain; Department of Internal Medicine, Hospital Universitario Miguel Servet, Zaragoza, Spain; Department of Internal Medicine, Hospital Clínico Universitario de SAntiago de Compostela, La Coruña, Spain; Rheumatology Department, Clínica Universitaria Bolivariana, Universidad Pontificia Bolivariana, Medellín, Colombia; Department of Internal Medicine, Hospital General Universitario Morales Meseguer, Murcia, Spain; Department of Internal Medicine, Hospital Universitario Vall d’Hebron, Barcelona, Spain; Department of Internal Medicine, Hospital Universitario Vall d’Hebron, Barcelona, Spain; Department of Internal Medicine, Hospital Universitario La Paz, Madrid, Spain; Department of Internal Medicine, Hospital Universitario La Fé, Valencia, Spain; Rheumatology Department, Hospital General de Medellín, Medellín, Colombia; Vanderbilt University, Nashville, Tennessee, USA; Department of Internal Medicine, Hospital Clínico Universitario Lozano Blesa, Zaragoza, Spain; Department of Internal Medicine, Hospital Clínico Universitario Lozano Blesa, Zaragoza, Spain; Department of Internal Medicine, Hospital Clínico Universitario Lozano Blesa, Zaragoza, Spain; Department of Internal Medicine, Hospital Universitario Miguel Servet, Zaragoza, Spain; Ingeniero Informático, Universidad de Zaragoza, Zaragoza, Spain; Department of Internal Medicine, Hospital Clínico Universitario Lozano Blesa, Zaragoza, Spain

**Keywords:** nailfold capillaroscopy, systemic sclerosis, disease pattern, examiner consensus, software-based analysis, quantitative analysis, CatBoost algorithm, machine learning-based model

## Abstract

**Objectives:**

Nailfold videocapillaroscopy (NVC) is the gold standard for diagnosing SSc and differentiating primary from secondary RP. The CAPI-Score algorithm, designed for simplicity, classifies capillaroscopy scleroderma patterns (CSPs) using a limited number of capillary variables. This study aims to develop a more advanced machine learning (ML) model to improve CSP identification by integrating a broader range of statistical variables while minimizing examiner-related bias.

**Methods:**

A total of 1780 capillaroscopies were randomly and blindly analysed by three to four trained observers. Consensus was defined as agreement among all but one observer (partial consensus) or unanimous agreement (full consensus). Capillaroscopies with at least partial consensus were used to train ML-based classification models using CatBoost software, incorporating 24 capillary architecture-related variables extracted via automated NVC analysis. Validation sets were employed to assess model performance.

**Results:**

Of the 1490 capillaroscopies classified with consensus, 515 achieved full consensus. The model, evaluated on partial and full consensus datasets, achieved 0.912, 0.812 and 0.746 accuracy for distinguishing SSc from non-SSc, among SSc patterns, and between normal and non-specific patterns, respectively. When evaluated on full consensus only, accuracy improved to 0.910, 0.925 and 0.933. CAPI-Detect outperformed CAPI-Score, revealing novel capillary variables critical to ML-based classification.

**Conclusions:**

CAPI-Detect, an ML-based model, provides an unbiased, quantitative analysis of capillary structure, shape, size and density, significantly improving capillaroscopic pattern identification.

Rheumatology key messages

- Machine learning improves accuracy in diagnosing SSc capillaroscopic patterns by eliminating examiner bias.
- CAPI-Detect classifies SSc patterns using 24 quantitative capillary variables with high reliability.
- The algorithm enables accessible, explainable and time-efficient classification for clinicians.

## Introduction

SSc involves early microvascular changes with endothelial cell dysfunction. There is a pressing need for routine, easily available procedures to monitor and assess microvascular damage closely. Nailfold videocapillaroscopy (NVC) allows for a non-invasive diagnosis of abnormalities in capillary architecture and is among the criteria established by the ACR/EULAR to determine SSc patterns [1]. Capillaroscopy is standardized for the analysis of microvasculature in the eight fingers of both hands, excluding the thumbs. Images can be obtained from the central millimeter or from up to four sequential 1-mm sections to create a panoramic view, ensuring a comprehensive assessment of capillary abnormalities. In recent years, significant progress has been made to exploit the potential of NVC. The so-called Fast Track algorithm, designed to identify scleroderma patterns, was developed as an easily manageable method accessible to capillaroscopists of any level of experience [2]. Additionally, given the time-consuming nature of visual examination of capillaroscopies, automated systems have been developed to study capillary images, facilitating the identification and classification of abnormalities [3–11].

Our group has been working on the development of simple procedures to identify SSc-patterns easily and promptly. Firstly, we created a deep learning–based software, trained with over 2500 manually labelled images, to obtain exhaustive NVC data without expensive equipment [12]. This software could individually identify giant capillaries, haemorrhages, tortuosities and abnormal capillaries [13], and assess capillary density. Subsequently, the external clinical validation was achieved by comparing algorithm predictions with annotations obtained by consensus of three or more out of five trained capillaroscopists on images from over 1000 patients with RP [14]. Recently, we leveraged our in-house-developed software to design an algorithm that assigns or discards SSc patterns using quantitative analysis of >22 000 NVC images from 881 SSc and RP patients. This algorithm, named CAPI-Score, discriminates between SSc and non-SSc patterns, stratifies SSc patterns into early, active or late according to Cutolo’s classification, and distinguishes between normal and non-specific patterns [15].

Nevertheless, hand-crafted algorithms have inherent limitations, such as failing to fully capture capillary architectural intricacies, potential selection bias and being too simplistic to utilize comprehensive numerical features. Machine learning (ML) can overcome these constraints by automatically selecting significant variables, thus achieving superior predictive performance [16, 17]. Given our software trained to identify and quantify capillary abnormalities and an extensive set of consensually agreed capillaroscopies from SSc and RP patients, we trained an ML-based model to assign DPs to capillaroscopies from patients with suspected or diagnosed systemic autoimmune disease. We hypothesized that this tool would perform predictions superior to manually designed algorithms and could be applied in routine clinical practice.

Our goal with CAPI-Score and CAPI-Detect is to design an algorithm that is able to reliably assign a pattern to a complete capillaroscopy, formed of at least one image per finger. The algorithm aims to achieve an accuracy comparable to that of an experienced capillaroscopist.

Importantly, neither algorithm is intended to predict or diagnose SSc or other illnesses, since an SSc capillaroscopic pattern does not always correlate with SSc. Therefore, the algorithm scope is limited to assigning patterns to capillaroscopies based on quantitative metrics. While the main goal is to assign an SSc or non-SSc pattern to the capillaroscopy with high accuracy, assigning a pattern for the SSc stage and assigning a normal *vs* non-specific pattern is also attempted by the algorithm.

## Methods

### Recruitment of patients for capillaroscopies collection with the aim to develop ML-based models to identify disease patterns

NVC images were taken in the course of routine explorations of patients with SSc or RP in nine Spanish and American hospitals (Supplementary Methods, available at *Rheumatology* online, see Data availability statement for access link). The Spanish researchers belonged to the Spanish Systemic Autoimmune Diseases Group (GEAS) and the Multidisciplinary Spanish Society of Systemic Autoimmune Diseases (SEMAIS). The following digital video capillaroscopes were used: Smart G-Scope (Genie Tech, Seoul, Republic of Korea); Optilia Digital Capillaroscope (Optilia Medical, Vällingby, Sweden); Inspectis Capillaroscope (Inspectis, Kista, Sweden); Dino-Lite CapillaryScope 200 PRO (Dino-Lite Europe, Almere, The Netherlands). A magnification of 200× was used in all cases.

The Capillary.io software, its database, and the project to build a ML-based algorithm to diagnose disease patterns, were approved by the Clinical Research Ethics Committee of Aragón (Spain), with record number PI18/336 (20/2018). Like in our previous projects involving NVC analysis by trained physicians and the software, patients signed an informed consent form where they agreed to participate. All the images were properly anonymized before their assignment to expert capillaroscopists.

### NVC analysis by trained specialists to set up training and validation sets

Nine capillaroscopists with large experience in NVC analysis manually examined the NVC images and followed EULAR criteria to classify them into one of the following categories [18]: normal; non-specific or SSc-early; SSc-active; and SSc-late according to Cutolo’s classification. Capillaroscopists were also permitted to classify a capillaroscopy as ungradable, either due to insufficient image quality or because a definitive assessment could not be established. Disease patterns were classified according to the examiner’s criteria, which were based on NVC hallmarks related to capillary density, presence of giant capillaries and/or haemorrhages, tortuosities or other abnormalities [14, 15].

Each capillaroscopy was randomly assigned to at least three, and at most four, of the nine examiners until completing the provisioned pool of work, who were blinded regarding the patient’s diagnosis. Examiners were instructed to classify the complete capillaroscopy into a specific pattern, with access only to the full set of images comprising the capillaroscopy. Partial consensus was deemed to occur when at least two out of three (≥2/3) or three out of four (≥3/4) examiners agreed on the pattern classification for a single capillaroscopy. Full consensus was considered when there was agreement among all examiners (3/3 or 4/4). Consensus among examiners would constitute the gold standard (real pattern) in later steps either to train ML-based models or to evaluate the performance of the trained models. Capillaroscopies deemed ungradable by consensus were also discarded. With this approach our goal was to train models that could classify capillaroscopies into established patterns at a level of accuracy similar to or better than that of a trained capillaroscopist focusing only on cases where expert classification is achievable. Determining whether a capillaroscopy was unclear or of poor quality was not an objective of the algorithm.

### Software-based analysis of capillaroscopies

Recruited capillaroscopies were then analysed using our in-house-developed software, Capillary.io, which has been developed according to accepted criteria to identify capillary abnormalities [18, 19]. Details on this tool, which has been comprehensively described and validated elsewhere [12, 14, 15], are provided in the Supplemental Methods (see Data availability statement for access link).

To guarantee that a capillaroscopy is complete, a minimum of eight images—one per finger—is required. Those capillaroscopies with fewer than eight images were discarded for software analysis. The analysis returned the results of each patient’s capillaroscopy as an aggregated statistic of the measured variables.

All software-generated variables belong to the following categories: total capillary density, density by type of capillary/haemorrhage, percentage distribution of each capillary type, capillary and haemorrhage area measurements, and statistical metrics of capillary loops and limbs categorized by capillary type.

Statistics for ML model training were obtained by aggregating the total counts of detected capillaries and haemorrhages across all analysed images. The total nailfold section length (in millimeters) was determined by summing the lengths from each individual image. Capillary and haemorrhage densities were then calculated by dividing the total counts for each type by the total analysed length. Additionally, global percentages were calculated to provide a comprehensive overview of each capillary type.

### Development of ML models to identify capillaroscopy patterns

When training an ML model with numerous variables, such as in this scenario, the final model may rely on highly complex decision rules derived from the training data, potentially leading to overfitting of the training set. Consequently, the model's performance may diminish when applied to real-world examples. To mitigate the risk of overfitting, the training process must be carefully designed [20]. In this case, a typical strategy was employed:

- The number of input variables used was limited to those most useful during the initial training. While the software is capable of producing variables aggregated globally and by finger, along with additional statistics such as minimum, maximum and s.d., we found that the model did not require a large number of variables to achieve good performance. As a result, we opted to retain only the global average aggregations for the final training.
- The dataset underwent division into a training set (80% of data) and a validation set (20%). Notably, only the latter was employed to generate the reported results, ensuring utilization of data unseen by the model during training.

We trained the CAPI-Detect models using a dataset of capillaroscopies classified as having either partial or full consensus on their disease patterns. After training, we evaluated the model’s performance on two validation sets: one that included both partial and full consensus classifications, and another limited to cases with full consensus only. This approach allowed us to compare the model’s performance on a larger but potentially noisier validation set (partial and full consensus combined) against a smaller, but more reliable validation set (full consensus only), providing insights into how label quality and dataset size affects model accuracy.

We selected CatBoost for training purposes, as it is a well-regarded, simple and efficient software package that trains highly accurate, fast, robust and powerful models for both numerical and categorical data. By sequentially building decision trees through a greedy search and ensembling techniques, CatBoost constructs a strong predictive model [21]. To optimize performance, we trained three distinct models, employing a two-stage approach. Initially, the first model differentiates between SSC and non-SSC patterns. Upon identification of an SSc pattern, a second model classifies the case into early, active or late patterns according to Cutolo’s classification. Conversely, if the result indicates a non-SSc pattern, the third model classifies the case into normal or non-specific patterns. This hierarchical method ensures that any given capillaroscopy can be assigned to a well-defined capillaroscopic pattern. This strategy mirrors the approach used in our previously described CAPI-Score algorithm [15], but it takes advantage of a machine learning framework that can incorporate a much larger set of variables, thereby improving accuracy. The final number of assessed variables was 24, enough to build reliable models without risking overfitting.

One of CAPI-Detect’s key advantages for assisting physicians is that it outputs pattern-specific probability scores. A lower probability for the predicted pattern indicates less confidence in the model’s prediction, whereas a higher probability suggests a more reliable classification. In cases where patients may be transitioning between patterns or when the situation is ambiguous, the probabilities for each pattern will be more evenly distributed, reflecting the model’s uncertainty in making a definitive classification.

### Assessment of CAPI-Detect models’ performance

The process explained above allowed us to create the CAPI-Detect algorithm, formed by three ML models. The next step was to assess the validity of these models. For this purpose, we evaluated them with the validation set that had been previously reserved. The results issued by the ML-created models (predicted patterns) were compared with those issued by the capillaroscopy specialists (real patterns). Precision/recall and receiver operating characteristic (ROC) curves were generated for these purposes. Proportion of matches and discrepancies between the gold standard classifications (examiners’ consensus) and the model’s predicted patterns were also calculated.

## Results

### Patients selected for training and validation sets

**Figure 1.**
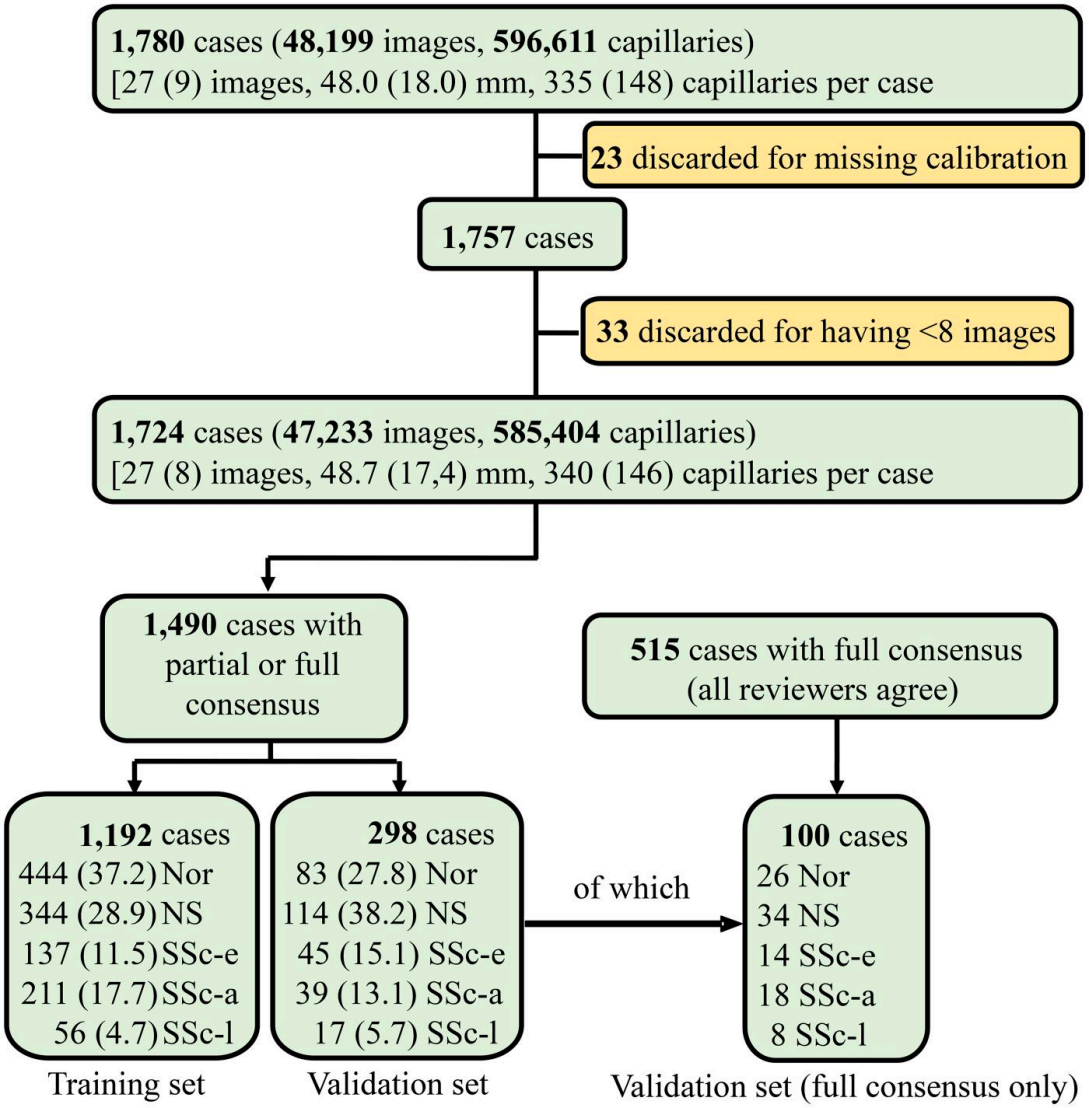
Flowchart diagram of the study. Capillaroscopies with partial and full examiner consensus (considered the gold standard) to be software-analysed and, subsequently, used to train the machine learning–based models. Approximately 20% of capillaroscopies were reserved as a validation set, not used for training. For additional evaluation, the validation set was restricted to cases with full examiner consensus only, allowing for an assessment of matches and discrepancies between manual analysis and the machine learning–based algorithm. This restriction ensured a focus on higher-quality cases with clearer classification among examiners. The percentage of each disease pattern in the training and validation sets is indicated in parentheses. Nor: normal; NS: non-specific; SSc-a: active scleroderma pattern; SSc-e: early scleroderma pattern; SSc-l: late scleroderma pattern

Initially, 1781 capillaroscopies were obtained from patients with SSc or RP, of which 1724 fulfilled all requirements to be considered valid for manual and software analysis (Fig. 1). Partial and full consensus was achieved in 1490 and 515 capillaroscopies, respectively. Normal, scleroderma and non-specific patterns were well represented in both groups, well above 425 and 125 cases each in the partial and full consensus cohorts, respectively (Supplementary Table S1, available at *Rheumatology* online).

### Development of CAPI-Detect models

From the consensus dataset, 298 capillaroscopies (∼20% of the total) were randomly selected as a validation set. These cases were not used for training but were reserved to evaluate the final models’ validity. The distribution of disease patterns was comparable between the training and validation sets (Fig. 1).

**Figure 2.**
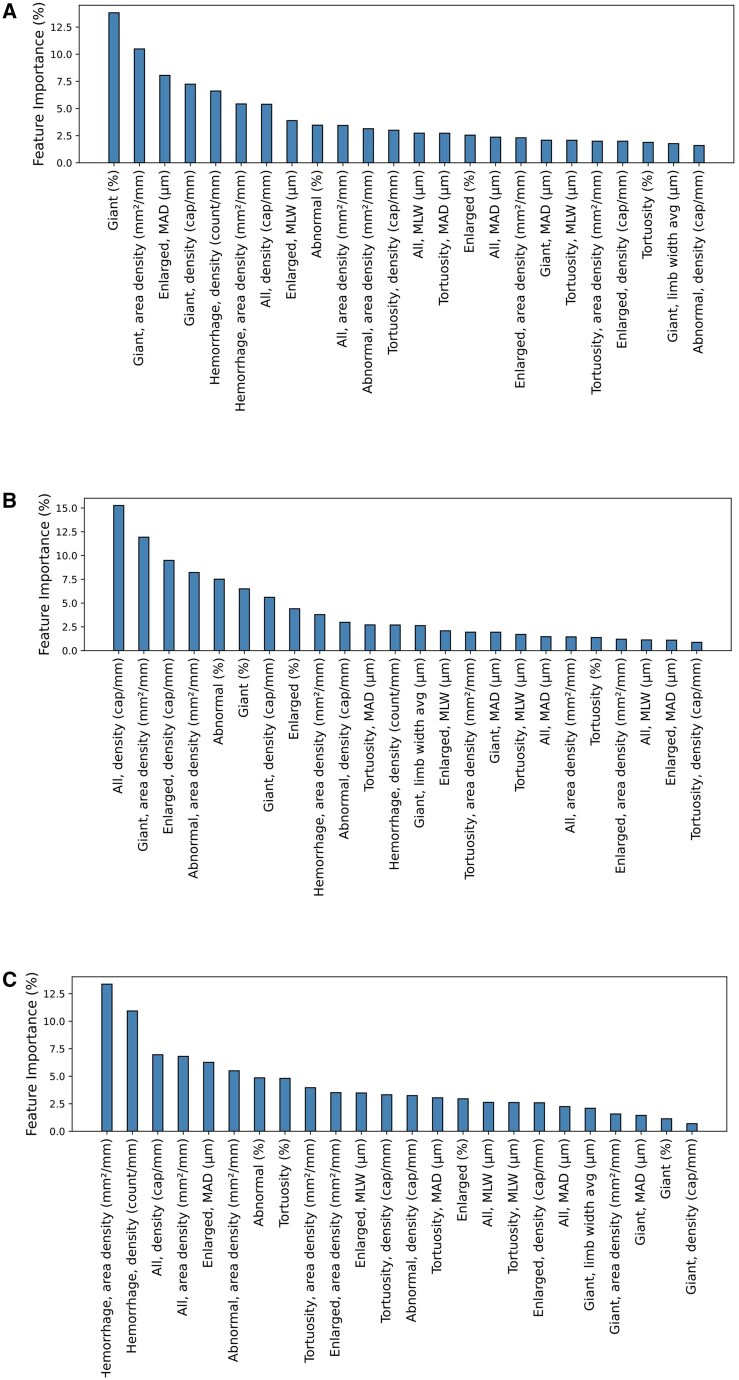
Weight of the software-assessed variables on the machine learning–based models. The weights of each variable for the three models that form the algorithm are shown for the steps to discriminate between non-SSc and SSc pattern (**A**), between early, active and late within SSc patterns (**B**), and between normal and non-specific within non-SSc patterns (**C**). Variables are arranged in decreasing order of weight. MAD: mean apical diameter; MLW: mean limb width

Fig. 2 illustrates the relative importance weight of each software-assessed variable in the final CAPI-Detect models. Notably, the top four variables collectively account for nearly 40% of the decision weight, with further details provided in Supplementary Figs S1–S3, available at *Rheumatology* online. These figures illustrate their distribution of key variables according to disease patterns assigned by partial and full consensus among examiners. Capillary density, giant capillary-related variables and mean capillary apical diameter were most influential to distinguish between SSc and non-SSc pattern (Fig. 2 and Supplementary Fig. S1, available at *Rheumatology* online). Variables related to abnormal capillaries, including the density of enlarged and total capillaries, as well as the area of giant capillaries were important to stratify SSc patterns into SSc-early, SSc-active or SSc-late (Fig. 2 and Supplementary Fig. S2, available at *Rheumatology* online). Finally, haemorrhages, along with the density and area of all capillaries, played a pivotal role in distinguishing normal from non-specific patterns (Fig. 2 and Supplementary Fig. S3, available at *Rheumatology* online).

### Performance metrics of CAPI-Detect

**Figure 3.**
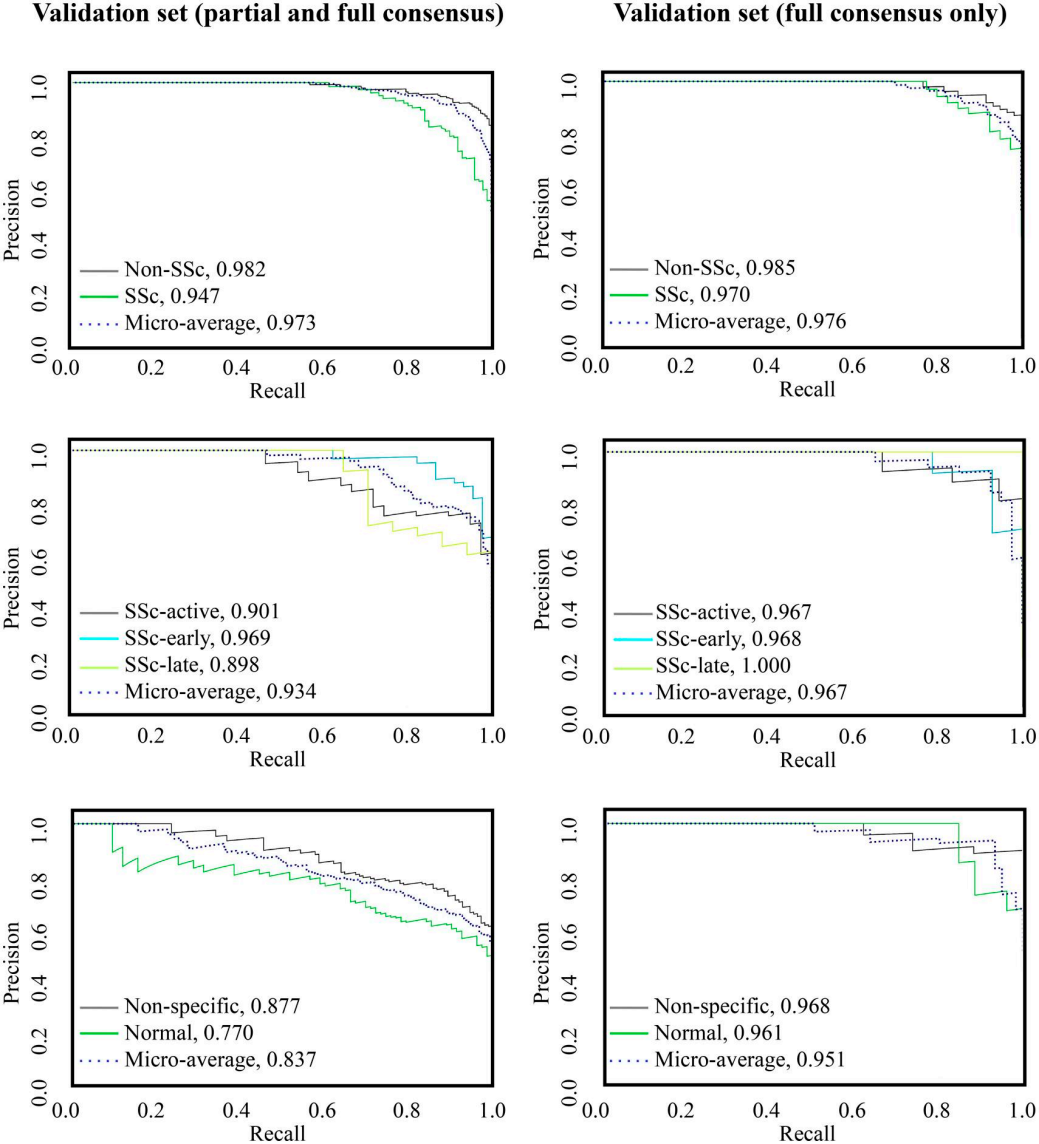
Precision-recall curves for the trained models. Precision-recall curves evaluated the accuracy of trained models using the validation set, and the validation set limited to full consensus cases, which consisted of 298 and 100 randomly selected capillaroscopies, respectively. AUPRC values corresponding to each disease pattern are shown. AUPRC: area under the precision-recall curve

The validation set capillaroscopies were analysed with the trained models, allowing us to calculate key performance metrics—precision, recall, and accuracy—at each decision point. These included: (i) distinguishing between non-SSc and SSc patterns; (ii) differentiating normal from non-specific patterns within the non-SSc group; and (iii) classifying SSc patterns into early, active, or late (Table 1). When evaluated on the combined partial and full consensus validation set, the model’s area under the precision-recall curve (AUPRC) reached 0.973 for distinguishing non-SSc *vs* SSc, 0.934 for classifying SSc subtypes, and 0.837 for separating normal *vs* non-specific patterns. Performance further improved when testing on the full consensus-only dataset, increasing the average AUPRC by 0.003, 0.033 and 0.114 for the three comparisons, respectively (Fig. 3). The ROC curves (false-positive rate *vs* true-positive rate) were also calculated for the SSc *vs* non-SSc pattern and, within the latter, for the normal *vs* non-specific pattern. The area under the ROC curves when comparing non-SSc *vs* SSc patterns were similar for partial and full consensus (0.97 *vs* 0.98, respectively), but significantly better when analysing non-specific *vs* normal (0.83 *vs* 0.97, respectively) (Supplementary Fig. S4, available at *Rheumatology* online).

**Table 1.** Performance metrics of the machine learning-built models to discriminate among disease patterns

Disease patterns to discriminate between	Partial and full consensus validation set	Full consensus only validation set				
	Recall (sensitivity)	Precision (PPV)	Accuracy	Recall (sensitivity)	Precision (PPV)	Accuracy
Non-SSc *vs* SSc		0.91		0.91		
Non-SSc	0.95	0.92		0.90	0.95	
SSc	0.84	0.89		0.93	0.86	
SSc-early *vs* SSc-active *vs* SSc-late			0.81		0.92	
SSc-early	0.91	0.87		0.86	0.92	
SSc-active	0.74	0.76		0.94	0.89	
SSc-late	0.71	0.75		1.00	1.00	
Normal *vs* non-specific		0.75		0.93		
Normal	0.71	0.66		1.00	0.85	
Non-specific	0.77	0.81		0.89	1.00	

The three trained models relied on machine learning-based algorithms. These were created after undergoing a training process using a training set consisting of randomly selected disease pattern-assigned capillaroscopies with partial and full consensus among examiners. Two randomly selected validation sets of 298 (partial and full consensus cases) and 100 capillaroscopies (full consensus cases only), were used to evaluate the performance of the models. PPV: positive predictive value.

**Figure 4.**
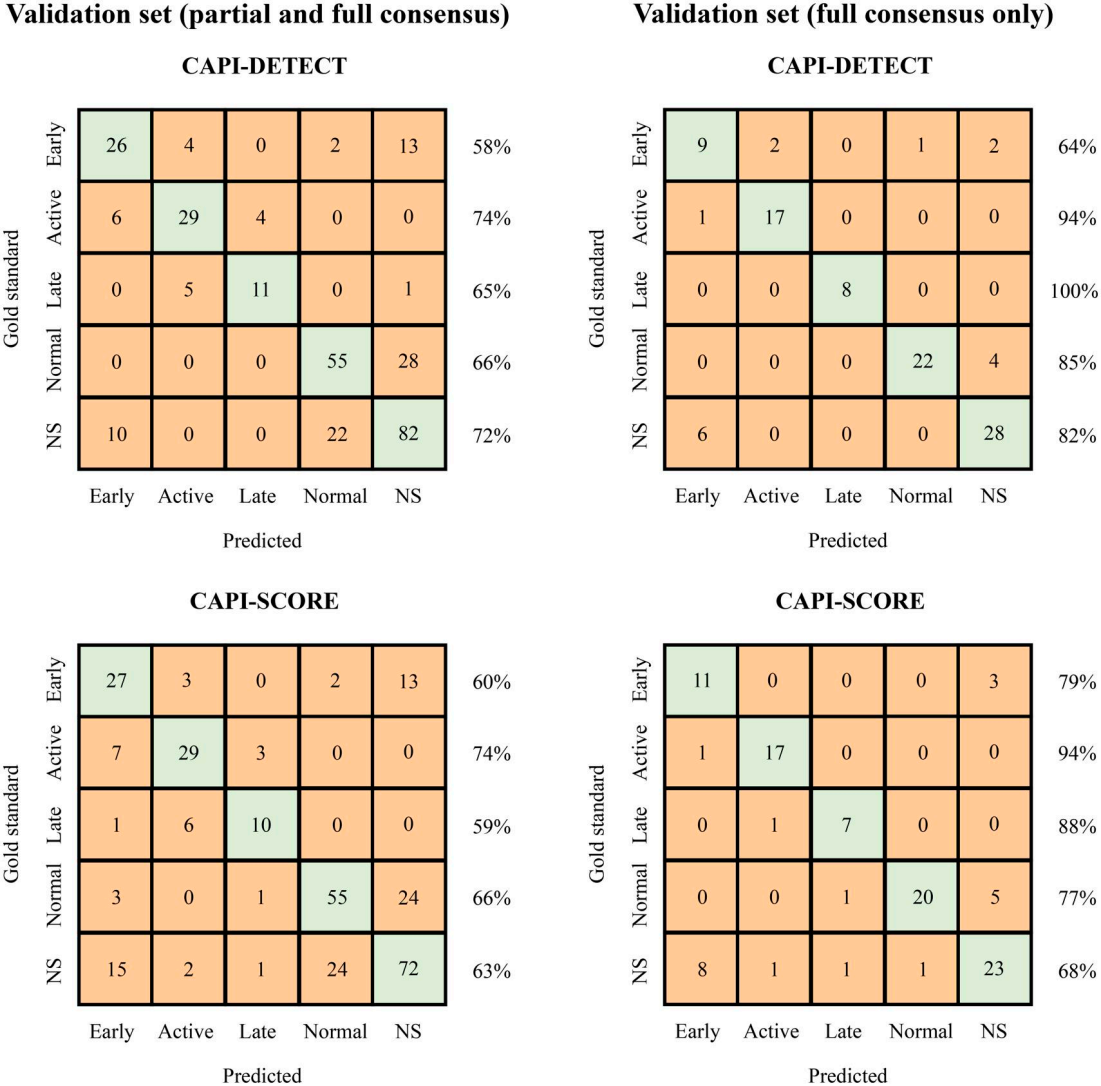
Confusion matrix comparing CAPI-Detect and CAPI-Score algorithm performance on the validation set for five-way pattern classification task. Two validation sets were assembled by randomly selecting 298 and 100 capillaroscopies with partial and full consensus, and full consensus only among examiners, respectively. Performance of machine learning-based CAPI-Detect and hand-crafted CAPI-Score algorithms for the five-way classification task [normal, non-specific (NS), early, active and late patterns] was evaluated with the validation sets. Confusion matrices display the performance of both algorithms. Accuracy for each pattern is indicated in percentages on the right-hand side of each matrix row

In order to validate the results of ML-based models against a hand-crafted algorithm, performance metrics for the five-way classification task (distinguishing among normal, non-specific, early, active and late patterns) were evaluated for both CAPI-Detect and CAPI-Score algorithms using partial and full consensus validation sets. The general accuracy of CAPI-Detect was superior to CAPI-Score, especially when classifying normal *vs* non-specific patterns (Fig. 4).

Finally, the accuracy for distinguishing SSc from non-SSc patterns was 84% on the partial consensus dataset and 95% on the full consensus dataset. Furthermore, when evaluated only on full consensus cases, the distribution of correct matches was more balanced (93% and 90% for SSc and non-SSc, respectively), indicating improved reliability in classification (Supplementary Fig. S5, available at *Rheumatology* online).

## Discussion

NVC is an important diagnostic tool in the work-up for SSc and identifying its disease patterns. Several tools and algorithms have been invaluable, significantly enhancing both manual and software-assisted NVC examination [2, 5, 15]. These methods have played a crucial role in our diagnostic processes. While recognizing their significant contributions, it is important to acknowledge the potential for researcher-related bias [22, 23] and the reliance on a limited number of variables in current diagnoses. Considering the complexity of capillary architecture, exploring additional elements not previously considered could offer an opportunity to further improve diagnostic accuracy. ML-based methods may be useful to achieve this goal.

Researcher-related bias can be minimized by selecting, for training purposes, those capillaroscopies classified with partial and full consensus among expert examiners. Remarkably, the number of variables to include without overfitting risk remains higher than those used in non-ML NVC interpretations reported previously, even though these were based on convolutional neural networks [2, 6–8, 10–12, 14, 15, 23–26].

In the ML field, the problem is known as a classification problem. The CAPI-Detect algorithm classifies patient’s NVC patterns using numerical variables generated by foundational object detection models, which comprehensively analyse capillaries and haemorrhages in each image. This approach avoids the need to process image data directly for pattern classification, allowing standard algorithms like decision trees to be employed. The use of CatBoost enabled us to build accurate, efficient models with clear, interpretable criteria [21].

ML models that are based on numerical variables, can benefit from better explainability than purely image-based models, as we demonstrated by exposing what variables were found to be most significant for pattern classification. As a result, the CAPI-Detect algorithm outputs not only the final decision probability but also the importance weight of the variables influencing a particular decision, providing useful information for the physician to understand why a capillaroscopy is classified into some pattern by the algorithm.

Researchers' expertise was demonstrated by the fact that >86% of capillaroscopies reached at least partial consensus, and nearly a third achieved full consensus. Furthermore, all disease patterns were well represented in the final cohort, and similarly distributed between training and validation sets, ensuring robust model evaluation. To optimize performance, we developed three distinct models, each operating in two stages. This approach facilitated the classification of any NVC into a specific capillaroscopic pattern, following a methodology similar to the construction of the CAPI-Score algorithm [15], but with the advantage of utilizing a broader range of variables, enhancing diagnostic accuracy.

Avoiding time-consuming manual pattern classification and achieving higher accuracy are not the only advantages of this procedure. CAPI-Detect has revealed previously unaddressed variables related to capillary architecture that may hold clinical significance. Our findings align with pivotal descriptions of disease patterns [27] and underscore the importance of well-established variables such as capillary density or giant capillaries in distinguishing between SSc and non-SSc [2]. Nevertheless, the predictive value of giant capillaries goes beyond the mere distinction between presence *vs* absence, their percentage, density, mean limb width (MLW) and mean apical diameter (MAD) each independently contribute to more accurate pattern identification. Furthermore, the MAD of enlarged capillaries, shorter in non-SSc patterns, is almost as influential as density. Enlarged capillaries are similarly influential, corroborating earlier associations with cutaneous involvement of SSc and DM [28, 29]. On the other hand, the proportion of abnormal capillaries appears useful for further stratifying SSc patterns, with little or rich presence associated with SSc-early or SSc-late pattern, respectively. Large density of enlarged capillaries and large area of giant capillaries are hallmarks of SSc-early and SSc-active patterns, respectively. Indeed, all capillary density is highest in the SSc-early and lowest in the SSc-late pattern. Finally, haemorrhages prove pivotal for discriminating normal from non-specific patterns.

Despite these advances, some limitations remain. Model training leaned on subjectively classified capillaroscopies, although limiting the training set to those capillaroscopies classified with at least partial consensus reduced the risk of learning from incorrect or ungradable capillaroscopies. The number of SSc-late pattern cases was relatively limited, but we still identified reliable diagnostic hallmarks. Moreover, the clinical relevance of non-SSc non-specific patterns remains uncertain.

In summary, our ML-based algorithm represents a powerful approach in identifying SSc patterns through comprehensive quantitative architectural variables. Our method enables more accurate classification when compared with hand-crafted algorithms, mitigating the risk of examiner-related bias and ensuring consistent results regardless of the examiner’s expertise level, all while saving time. Furthermore, our procedure opens avenues for objectively and reliably identifying new clusters, potentially enhancing the information gleaned from NVC examination. This may provide valuable insights into disease progression mechanisms, facilitating better understanding and management of SSc. The usefulness of nailfold capillary-based methods for predicting architectural changes in other autoimmune diseases requires further research, and methods like ours may help better characterize novel capillaroscopic patterns in these clinical scenarios.

## Supplementary Material

keaf073_Supplementary_Data

## Data Availability

Data and source code will be made available publicly after paper publication in the following online location: https://github.com/capi-detect. The complete supplementary methodology behind this study can be accessed via: https://drive.google.com/file/d/1yTTtmYxuMYDvhqY8KSTv_xRsv5qRzSog/view.
