# Supplementary material for: CAPI-Detect: machine learning in capillaroscopy reveals new variables influencing diagnosis
Source: Rheumatology (Oxford). 2025 Feb 7;64(6):3667–75. doi: 10.1093/rheumatology/keaf073 (PMC12107046; doi:10.1093/rheumatology/keaf073)
Supplement: keaf073_Supplementary_Data [file keaf073_supplementary_data.docx]

**Supplementary Figure S1. Disease Pattern distribution: Key variables for discriminating SSc from Non-SSc patterns**
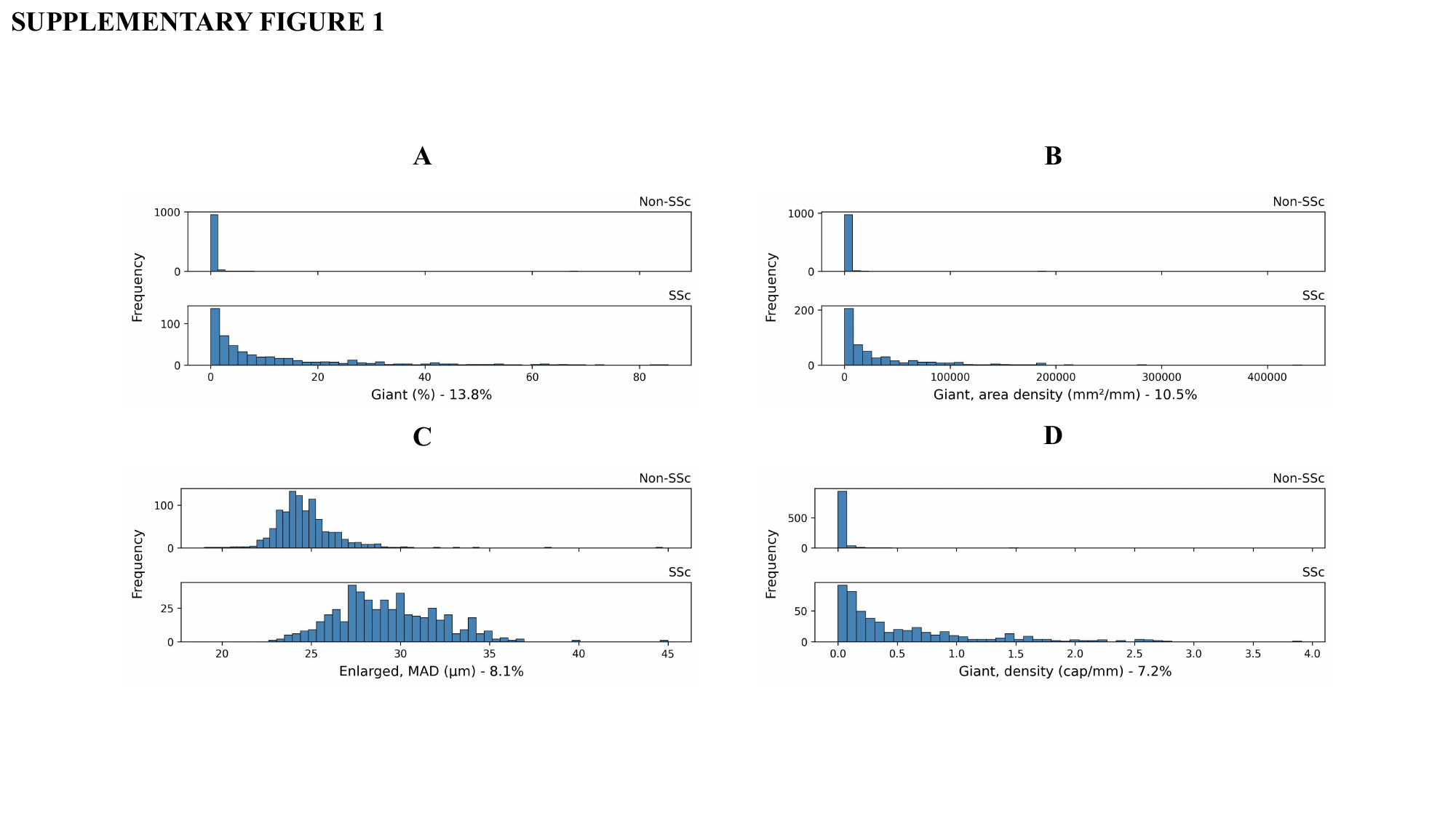


The percentage of decision weight to categorize disease patterns as SSc or non-SSc in the model trained to discriminate SSc from Non-SSc is indicated for each variable on the X-axis.

MAD, mean apical diameter; SSc, scleroderma.

**Supplementary Figure S2. Disease Pattern distribution in capillaroscopies categorized within the SSc group: Emphasizing four variables with the greatest decision weight for discriminating among Early, Active, and Late patterns**


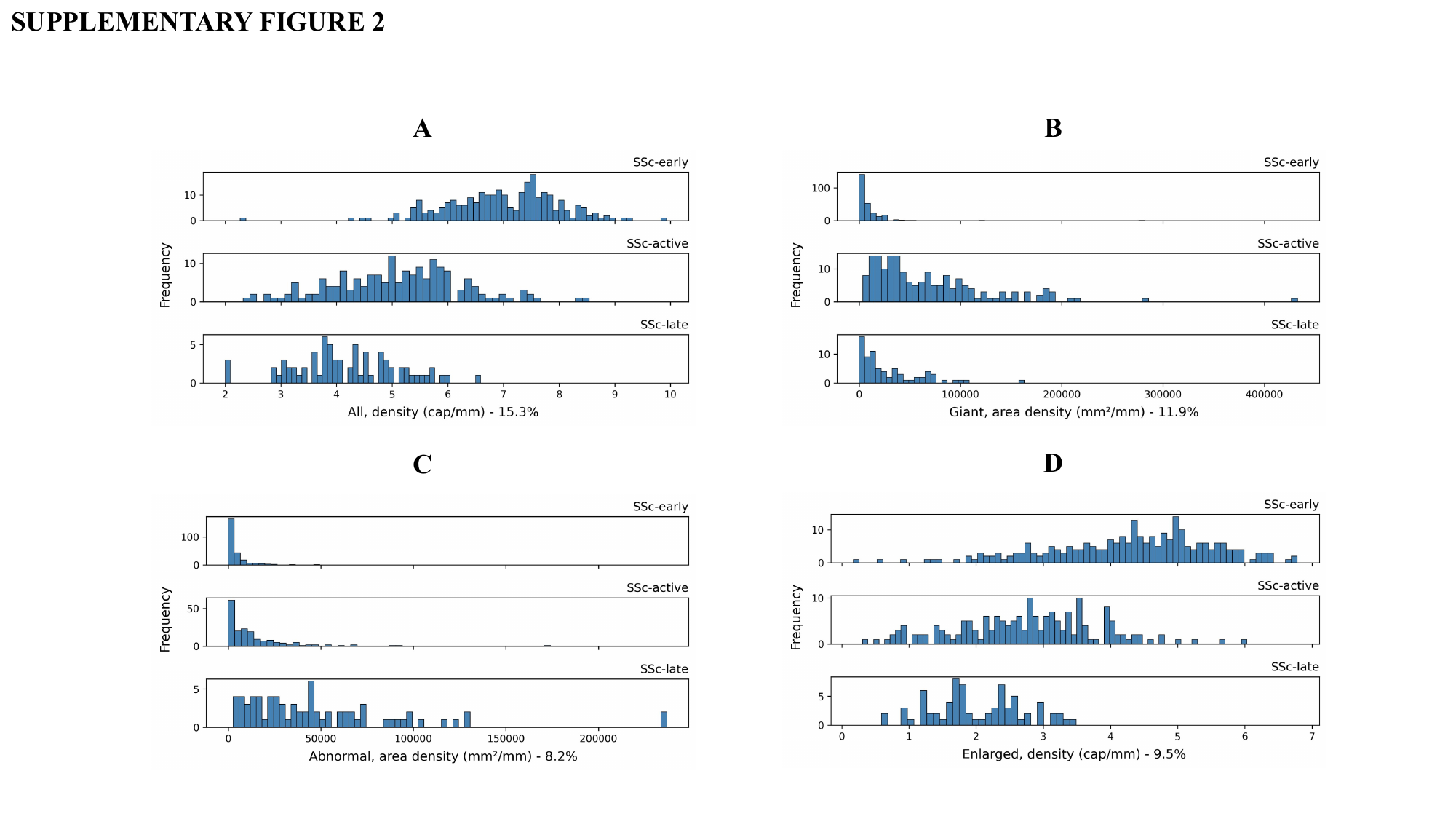


The percentage of decision weight to categorize SSc disease patterns as SSc-early, SSc-active or SSc-late is indicated for each variable on the X-axis.

SSc, scleroderma.

**Supplementary Figure S3. Disease Pattern distribution in capillaroscopies categorized within the Non-SSc Group: Focusing on variables with highest decision weight for discrimination between Normal and Non-Specific patterns**

**
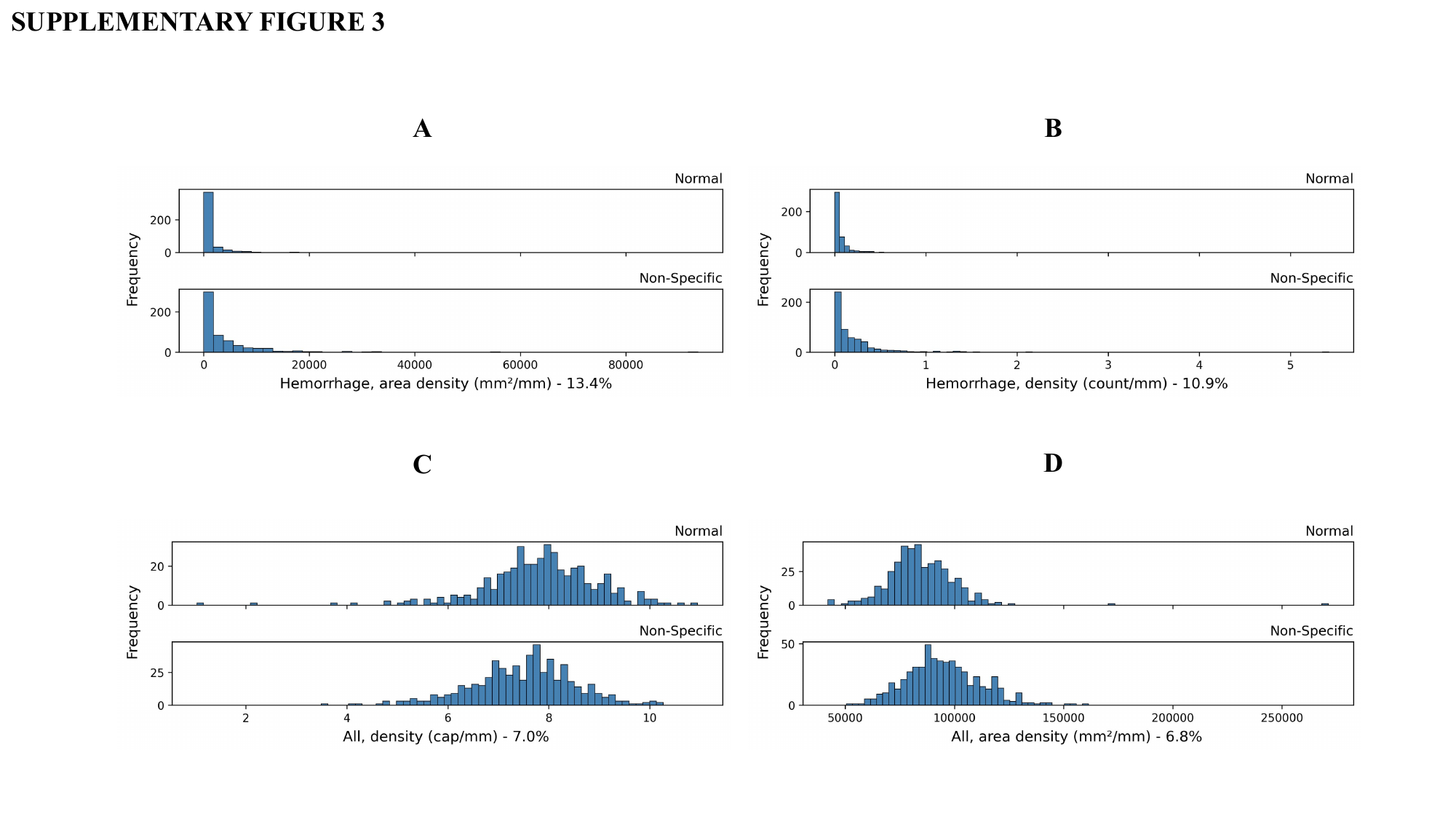
**

The percentage of decision weight to categorize non-SSc disease patterns as normal or non-specific is indicated for each variable on the X-axis.

SSc, scleroderma

**Supplementary Figure S4. Receiver operating characteristic curves**

**
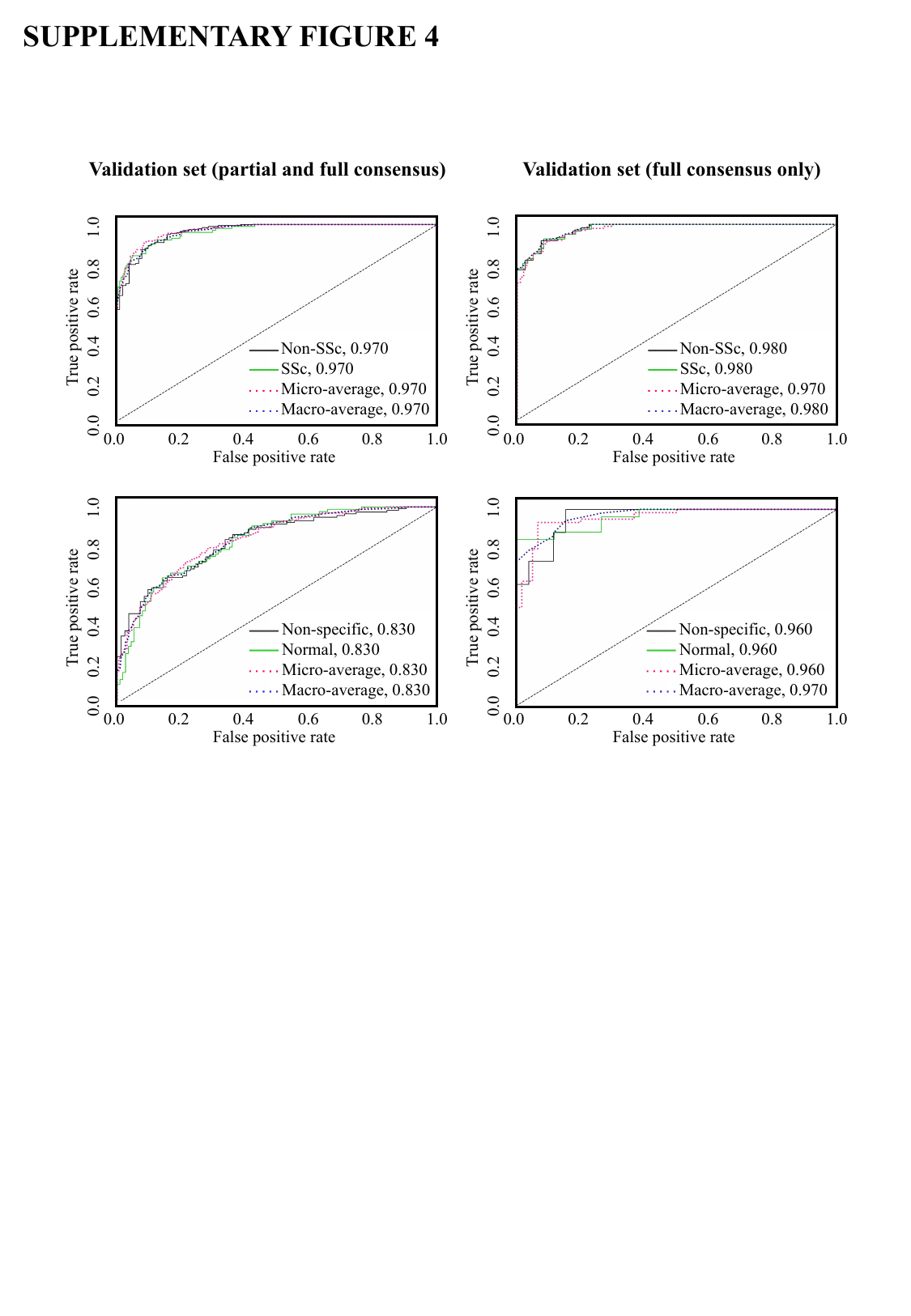
**

ROC curves further evaluated the accuracy of the 3 trained models, evaluated with the validation set (left panels) and full consensus only validation set (right panels), which consisted of 298 and 100 randomly selected capillaroscopies. AUROC values corresponding to each disease pattern are shown.

AUROC, area under the receiver operating characteristic curve; ROC curve, receiver operating characteristic curve.

**Supplementary Figure S5. Confusion Matrix: Agreement and Discrepancies between true and predicted disease patterns for each separate classification model**
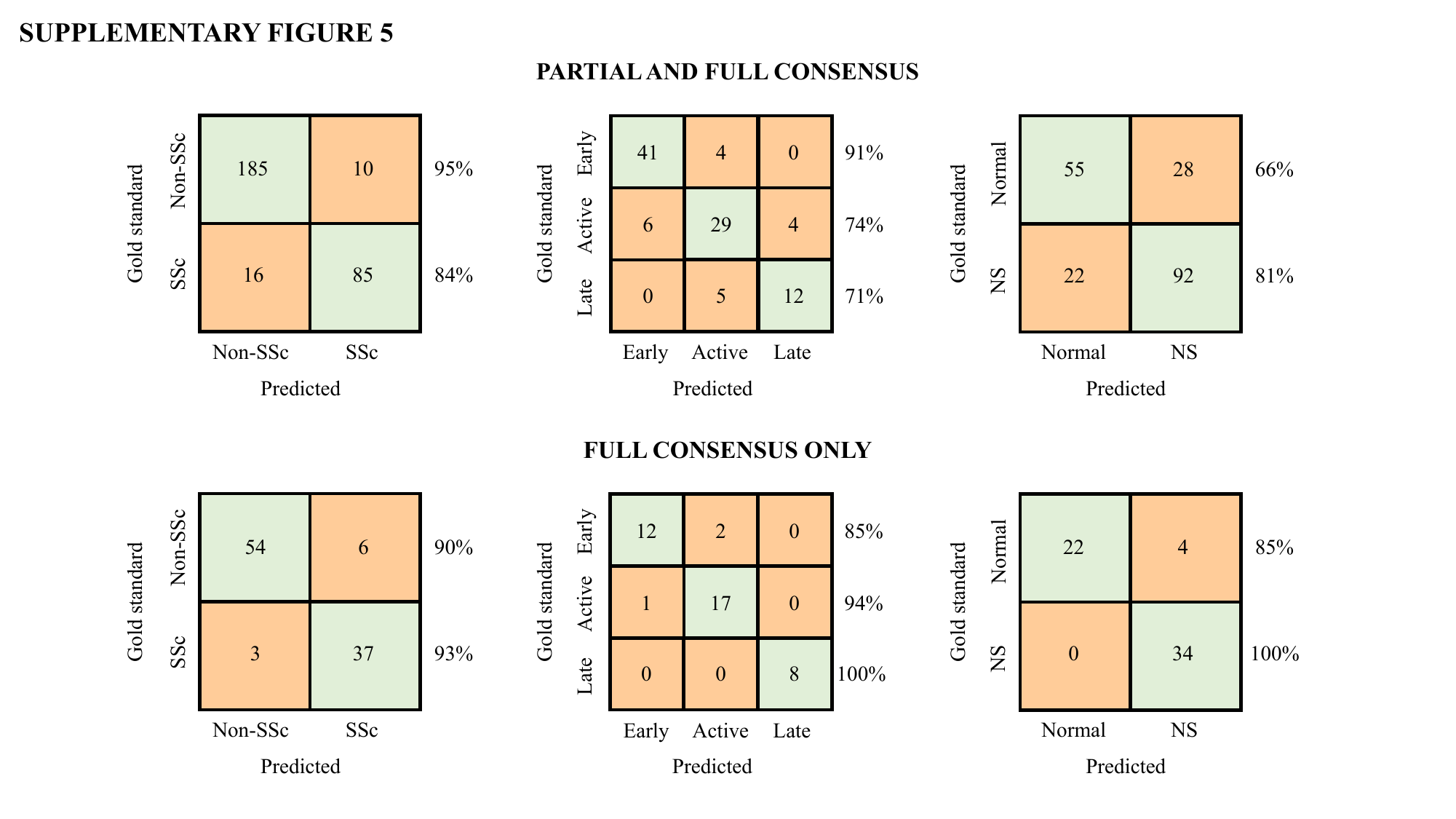


Two validation sets were assembled by randomly selecting 298 and 100 capillaroscopies with partial and full consensus, and full consensus only among examiners, respectively. Capillaroscopies were subsequently analyzed with the 3 trained models. Finally, the proportion of matches (green squares) and discrepancies (orange squares) in pattern assignment between examiner consensus (gold standard, real patterns) and machine learning-based models (predicted patterns) was reported for each model that composes the CAPI-Detect algorithm. Accuracy for each pattern is indicated in percentages on the right-hand side of each matrix row.

SSc, scleroderma

| **Supplementary Table S1** Consensus agreement among capillaroscopists for disease pattern identification of capillaroscopies | | |
| --- | --- | --- |
| **Analysis** | **Consensus** | |
|  | **Partial** | **Full** |
| Capillaroscopies analyzed, n | 1,724 | 1,724 |
| Capillaroscopies with consensus, n (%)* | 1,490 (86.4) | 515 (29.9) |
| Patterns with consensus reached, n (%)^†^ |  |  |
| Normal | 527 (35.4) | 154 (29.9) |
| Non-specific | 458 (30.7) | 145 (28.1) |
| SSc |  |  |
| Early | 182 (12.2) | 92 (17.9) |
| Active | 250 (16.8) | 84 (16.3) |
| Late | 73 (4.9) | 40 (7.8) |
| Consensus was considered to occur when all or all but one examiners agreed on pattern assignment. *Percentage according to total number of analyzed capillaroscopies. ^†^Percentage according to total number of capillaroscopies with consensus reached.  SSc, scleroderma. | | |
